# POCA: a CPG signal analysis algorithm using peak-based feature extraction and machine learning

**DOI:** 10.3389/fnins.2026.1740554

**Published:** 2026-03-11

**Authors:** Xu Han, Giuliano Taccola, Stanislav Culaclii, Atiyeh Mohammadshirazi, Yan-Peng Chen, Wentai Liu

**Affiliations:** 1 Department of Bioengineering, University of California, Los Angeles, Los Angeles, CA, United States; 2 Department of Neuroscience, International School for Advanced Studies (SISSA), Trieste, Italy; 3 Applied Neurophysiology and Neuropharmacology Lab, Istituto di Medicina Fisica e Riabilitazione (IMFR), Udine, Italy; 4 HRL Laboratories, LLC., Malibu, CA, United States; 5 Department of Electrical and Computer Engineering, University of California, Los Angeles, Los Angeles, CA, United States; 6 California NanoSystems Institute, University of California, Los Angeles Los, Angeles, CA, United States; 7 Brain Research Institute, University of California, Los Angeles, Los Angeles, CA, United States

**Keywords:** central pattern generator, feature extraction, fictive locomotor rhythm, machine learning, oscillation detection

## Abstract

Our understanding of the central pattern generator (CPG) for locomotion is primarily based on motor output analyses in isolated neonatal rodent preparations. Recent studies show that biomimetic neural modulation protocols, which mimic biological signals, outperform traditional methods in sustaining long-lasting fictive locomotor rhythms. However, fine-tuning such protocols requires extensive experimental trials, highlighting the urgent need for an automated CPG signal analysis tool. This study introduces the Peak-based Oscillation Classification Algorithm (POCA) for analyzing CPG signals using a novel peak-based feature extraction and machine learning. Although epoch-based feature extraction is widely applied in other biological oscillation analyses, they are suboptimal for CPG signals due to issue like challenging annotation and indirect feature representation. POCA addresses these limitations by extracting features directly from individual oscillation peaks, enabling more accurate and interpretable classification of locomotor versus non-locomotor activity. Using datasets from three independent stimulation protocols, a thresholding method using “peak prominence” feature achieved an F1 score of 0.911 and accuracy of 0.957, demonstrating the effectiveness of “peak prominence” as a key discriminative feature. A radial basis function kernel Support Vector Machine, incorporating additional peak features, further improved performance to an F1 score of 0.923 and accuracy of 0.966. The locomotor rhythm characterization results, based on oscillation detection, also aligned closely with human-expert assessments. The proposed POCA algorithm provides a robust, scalable tool for CPG signal analysis, facilitating large-scale evaluation of biomimetic protocols. The novel peak-based feature extraction framework also offers a versatile strategy for broader biological oscillation detection tasks.

## 1 Introduction

The locomotor central pattern generator (CPG) in the spinal cord is a remarkable neural network that can produce rhythmical motor output in the absence of continuous sensory input or precise external timing signals (Marder and Calabrese, 1996; Harris-Warrick, 2013). It activates the flexor and extensor motor pools for vertebrate locomotion (Gossard et al., 2010). Over the past decades, an increasing number of studies of spinal-cord electrical neuromodulation have successfully demonstrated the ability to induce CPG locomotor activity without descending commands, showing potential in assisting with neurorehabilitation for spinal cord injury (SCI)(Sayenko et al., 2022; Chalif et al., 2024).

In an *in-vitro* experiment, fictive locomotion (FL) can be evoked by stimulating dorsal root (DRs) with a train of stereotypic stimuli at a single frequency of 2–10 Hz (Marchetti et al., 2001). However, such trains of afferent stimuli induce only transient epochs of locomotor cycles. To generate a long-lasting FL activity, many novel, more effective modulation patterns were demonstrated (Taccola, 2011; Dose et al., 2013; Dose and Taccola, 2016). These include biomimetic neural stimulation using waveforms derived from ventral root (VR) motor outputs or human electromyography (EMG) recordings. Such biologically inspired patterns have shown enhanced CPG activation, even at lower amplitudes, and significantly broaden the design space for neural modulation. However, fine-tuning these protocols requires extensive experimental trials and detailed signal analysis. Currently, for each trial, experts manually identify locomotor-related oscillations from a sample VR and quantify rhythm metrics, such as the number of oscillations and rhythm duration, to assess stimulation effectiveness (Taccola, 2011; Dose et al., 2013; Dose and Taccola, 2016). This repetitive process is labor-intensive and impractical for large datasets. Furthermore, selecting an exemplar trace for each repetition lacks consistency in assessing the effects across all recorded VRs. This approach may overlook important variations and interactions that occur simultaneously across the full set of signals. To date, no specialized algorithms have been developed specifically for automated CPG signal detection and analysis, despite the importance of this task.

To address this gap, we first examine established methodologies in other biological oscillation detection domains, such as high frequency oscillation (HFO) detectors [10], and beta oscillation detector using electroencephalogram (EEG)[11]. These methods often rely on statistical thresholding, applying fixed criteria to short-time energy measures, such as root mean square, envelope, entropy, or line length, to detect oscillatory events (Le Van Quyen and Bragin, 2007; Zelmann et al., 2012; Karvat et al., 2020). Although conceptually simple, these methods are susceptible to false detections due to the non-Gaussian, non-stationary characteristics of energy measures and substantial inter-individual variability (Whitten et al., 2011; Janca et al., 2015; Navarrete et al., 2016). To overcome these limitations, recent studies have increasingly adopted data-driven strategies. Many machine learning approaches rely on an epoch-based feature extraction framework, where the continuous signal is segmented into fixed-length time epochs (Siddiqui et al., 2020). From each epoch, features are extracted to characterize the signal, typically from the time, frequency, or time-frequency domains (Boonyakitanont et al., 2020; Siddiqui et al., 2020). These features are then used to train classifiers such as linear discriminant analysis (LDA), support vector machines (SVMs), or neural networks (NNs) to predict the presence or absence of target oscillations (Siddiqui et al., 2020; Wang et al., 2022).

However, directly applying epoch-based frameworks to CPG analysis presents critical challenges. First, when multiple types of oscillations, such as non-locomotor oscillations and locomotor oscillations, coexist within a epoch, labeling each epoch becomes difficult. Second, segmenting the signal into epochs results in a limited number of mutually independent training samples, potentially requiring more experiment trials to obtain sufficient training size. Finally, because this framework extracts regional features from entire epochs rather than directly from individual oscillations, it provides a less precise representation of each oscillatory event, which can reduce the accuracy in distinguishing between locomotor and non-locomotor activity.

To address these limitations, we propose the Peak-based Oscillation Classification Algorithm (POCA), a novel signal analysis framework for CPG activity. Rather than adopting the epoch-based feature extraction approach commonly used in other applications, POCA introduces an intuitive peak-based framework integrated into a machine learning pipeline. It extracts 12 physiologically relevant features from each candidate oscillation peak within a reference window, including key local features such as *peak prominence* (reflecting oscillating intensity) and regional features such as *local deviation* (capturing contrast with neighboring peaks). This approach is particularly well-suited for CPG locomotor signals, capturing both local and regional characteristics essential for accurate classification. By applying machine learning classifiers at the peak level, POCA improves classification accuracy and reduces post-processing complexity, enabling precise characterization of locomotor rhythms. Moreover, it remains effective even with limited datasets, as each recording typically contains many independent oscillations, providing sufficient training samples. These design principles make POCA broadly applicable to biological oscillation detection tasks that require detailed local and regional signal features, especially under data-constrained conditions, and may offer promise in other applications such as HFO and seizure detection in EEG, where similar challenges in oscillation characterization exist. Most importantly, POCA offers an automated and robust solution specifically tailored for CPG signal analysis, facilitating the fine-tuning of biomimetic neural modulation protocols in large-scale experimental trials.

The paper is organized as follows. Section 2 introduces the proposed POCA algorithm, detailing the peak-based feature extraction framework and the machine learning pipeline. Section 3 describes the datasets and experimental setup used for evaluation. Section 4 presents the analysis of peak features and algorithm performance. Section 5 provides a detailed discussion and Section 6 concludes the paper. All the abbreviations mentioned in this paper are summarized in Supplementary Appendix 1.

## 2 Materials and methods

### 2.1 Overview

**FIGURE 1:**
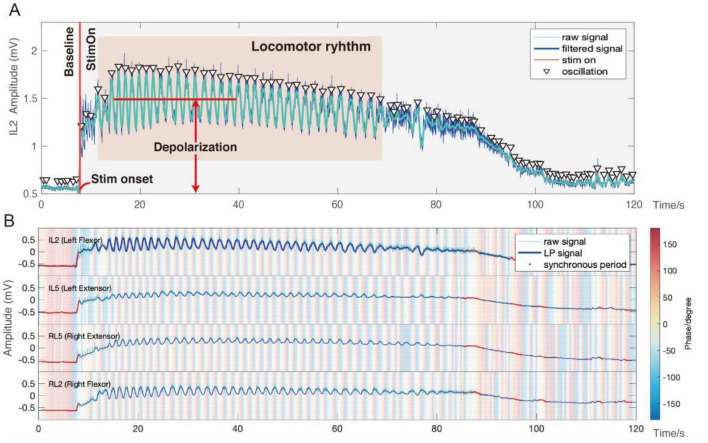
Illustration of a typical episode of electrically induced fictive locomotion. **(A)** CPG response of the flexor-related lumbar ventral root (VRlL2) to electrical stimulation at a sacrocaudal dorsal root. **(B)** A multi-channel phasic relationship demonstrating flexor-extensor coordination and the alternating left-right gait.

Like many neural signals, CPG signals contain segments with periodic amplitude oscillations (Le Van Quyen and Bragin, 2007). In the absence of sensory input or external stimulation, these oscillations occur spontaneously with a relatively low amplitude, known as the “baseline” period (Figure 1A). The baseline oscillations exhibit synchronous phase across channels (Figure 1B). When the spinal cord receives a sensory input or is subjected to electrical or chemical stimulation (Harris-Warrick, 2013), the period is referred to as the “StimOn” period (Figure 1A). A few seconds after the start of this period (“stim onset”), the signal undergoes a rapid “depolarization,” leading to intense locomotor oscillations with pronounced peak-to-peak amplitudes. At this stage, the CPG oscillations exhibit an out-of-phase relationship across channels, indicative of the flexor-extensor coordination and the alternating left-right gait, known as “locomotor rhythm” (Figure 1B). Eventually, the locomotor oscillations diminish and cease despite ongoing stimulation, with the signal return, or “repolarizing” to its baseline level.

The proposed POCA algorithm detects and analyzes the oscillations from CPG signal. It takes a *N*_*chan*_ channels signal **y**^(*c*)^ (*c* = 1,…,*N*_*chan*_, as the channel index) as input, detects all the oscillation peaks and characterizes each by *m* peak-features for each channel. Then all the oscillations are predicted as either true or false locomotor oscillations. Additionally, POCA calculates *k* rhythm-features to characterize the locomotor rhythm for each channel.

POCA processes the CPG signals **y**^(*c*)^ in five steps: pre-processing, synchronicity calculation, candidate oscillation detection, oscillation classification, and locomotor rhythm characterization. The algorithm pipeline is shown in Supplementary Appendix 2.

### 2.2 Pre-processing

To better prepare for the CPG oscillations detection, the raw signal **y**^(*c*)^ is first down sampled to 500 Hz, and then low pass filtered at 1.5 Hz (CPG locomotion bandwidth ranges from 0.15 to 1.5 Hz in this study) to produce the filtered signal ${\text{y}}_{\text{LP}}^{\left(c\right)}$. The CPG locomotion bandwidth might vary slightly across different experiments (Mor and Lev-Tov, 2007; Zhong et al., 2011; Harris-Warrick, 2013).

### 2.3 Cross-channel synchronicity analysis

During the locomotion, CPG signal generally exhibit an out-of-phase relationship among channels, also interpreted as an un-synchronous period across all channels. Thus, it is important to calculate the cross-channel synchronicity (Taccola, 2011; Dose et al., 2013) to confirm an a valid locomotor oscillation. To achieve this, the instantaneous phase signals ϕ^(*c*)^ of each channel is first estimated by applying Hilbert transformation H(⋅) and then extracting phase from the low-pass-filtered signal ${\text{y}}_{\text{LP}}^{\left(c\right)}$ (Blackledge, 2006), as Equation 1 shown:


$$\phi^{\left(c\right)}=\text{imag}\,\left(\log\text{H}\,\left({\text{y}}_{\text{LP}}^{\left(c\right)}\right)\right),c=1,\ldots ,{\text{N}}_{\text{chan}}$$ (1)

Next, the synchronicity signal ***s***^(*c*)^ is determined by Equation 2, where a synchronous state at each timepoint is defined when the phase difference between channel c and any other channels is < 45. Importantly, this analysis can be performed only when the data includes at least one pair of channels exhibiting a left-right or flexor-extensor alternating waveform.


$$s^{\left(c\right)}\left(t\right)=\left\{ \begin{array}{l} \text{True},\;\left|\phi^{\left(c\right)}(t)-\phi^{\left(i\right)}(t)\right|<{\text{45}}^{{}^{\circ}},\;\forall i\in \{1,\ldots ,{\text{N}}_{\text{chan}}\}\\ \text{False},\text{ otherwise} \end{array}\right.$$ (2)

### 2.4 Candidate oscillation detection and feature extraction

Before the oscillation classification process, all candidate oscillations are detected as the local maxima of the filtered signal ${\text{y}}_{L\,P}^{\left(c\right)}$. Then, each candidate is characterized by *m* peak-features (*m* =  12 in this study).

#### 2.4.1 Dynamic algorithm for estimating prominence and width

**FIGURE 2:**
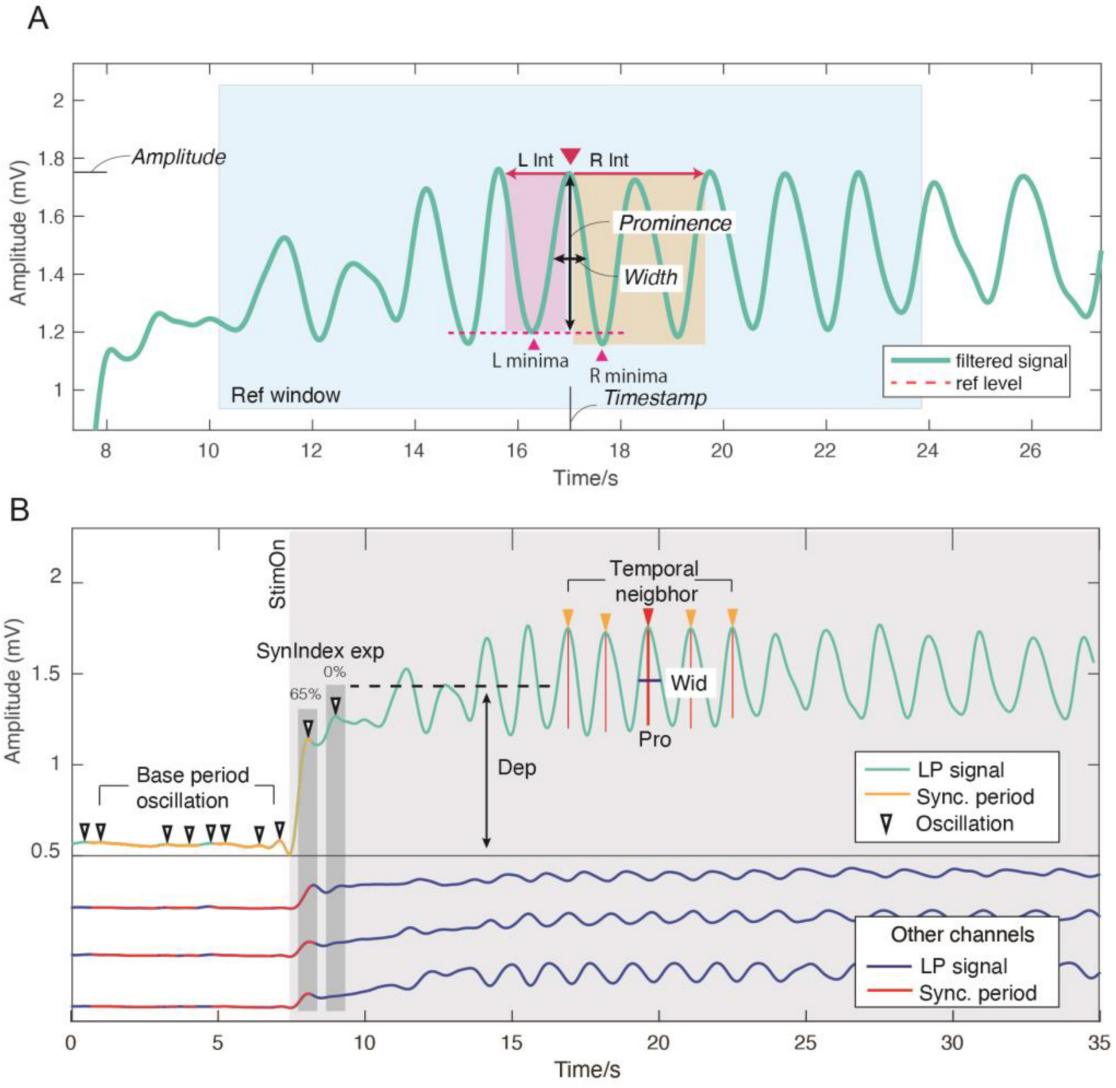
Peak-features extraction for candidate oscillation. **(A)** Calculation of prominence, width, amplitude, and timestamp; The red triangle indicated the peak under analysis; The blue widow indicated the reference window of the target peak; L/R int referred to Left/Right search interval; L/R minima referred to Left/Right minima; The dashed line indicated the reference level. **(B)** Illustration of oscillations in baseline period, depolarization level (“Dep”), temporal neighbor peaks, temporal width (“Wid”), and synchronicity index example (“SynIndex exp”).

Among 12 peak-features, there are four basic features, as shown in Figure 2A: time of the peak (“Time”), absolute amplitude of the peak (“Amp”), prominence of the peak which reflects the oscillating intensity (“Pro”), and temporal span or width of the peak (“Wid”).

Due to the non-stationary nature of CPG signal, a dynamic algorithm is proposed to estimate each peak’s prominence and width feature (“Pro” and “Wid”). To begin with, a reference window of +6.67s (±1/0.15 Hz) is defined around a peak under analysis as Figure 2A. Within this window, the nearest higher peaks on left/right are identified to define search intervals. The lowest point of the signal within each interval is designated as the interval minimum. The higher of the two minima is taken as the reference level, which is then subtracted from the peak amplitude to compute the peak’s prominence (“Pro”). The temporal width (“Wid”) is defined as the distance between the points where the signal reaches half of this prominence. Calculating prominence within a local stationary reference window allows for a more accurate and robust measurement of oscillation intensity, compared to using absolute amplitude or estimating prominence over the entire non-stationary signal.

#### 2.4.2 Extracting the remaining features

Besides the four basic features, eight additional features are proposed to characterize candidate oscillation from more perspectives (Figure 2B).

Among these, three relative-prominence features “Pro2Dep,” “Pro2Base,” “Pro2Wid” are proposed by comparing “Pro” to signal depolarization level, mean prominence of baseline-period oscillations, temporal width, respectively. “LocDev” is proposed to measure the deviation of candidate’s prominence to its four temporal neighbors (two before and two after, to ensure symmetric and locally contextual comparison, as empirically effective).

“SynIndex” measures the degree of synchronicity of a candidate oscillation with other channels. It calculates the percentage of True synchronous status (**s**^(*c*)^ = *True*) within the 0.4-s window around the candidate oscillation, where a larger value means the phase was more similar with the other channels. As shown in Figure 2B, the example waveform reaches a “SynIndex” of 65% at 8 seconds, corresponding to the onset of depolarization, and drops to 0% at 9 s, after the CPG establishes an alternating phase relationship.

Finally, three Boolean features are introduced: “StimCK” determines whether the oscillation happened after the stimulation onset, “FreqCK” determines whether the oscillation’s frequency stayed within locomotion bandwidth, and “nSynCK” determines whether the oscillation’s degree of synchronicity was smaller than 50% (non-synchronous). Supplementary Appendix 3 summarizes the definitions of all 12 proposed peak-features, which are automatically extracted from oscillations.

### 2.5 Classifying oscillations

After extracting the peak-features from candidate oscillations, three approaches (thresholding, SVM, and K-means) are tested as classification models in this study. The input is a feature matrix ${\text{X}}^{\left(c\right)}\in {\mathbb{R}}^{N_{o\,s\,c}^{\left(c\right)}\times m}$, where *m* represents the number of peak-features and $N_{o\,s\,c}^{\left(c\right)}$ denotes the number of oscillation candidates of channel c. The output is a prediction vector ${\text{p}}^{\left(c\right)}\in {𝔹}^{N_{o\,s\,c}^{\left(c\right)}}$, with each element indicating either True (locomotor oscillation) or False (non-locomotor oscillation), shown in Equation 3. All three classifiers are implemented with a custom MATLAB script using machine-learning toolbox (MATLAB, 2020).


$${\text{p}}^{\left(c\right)}=f\,\left({\text{X}}^{\left(c\right)}\right)$$ (3)

Approach A: thresholding on single peak-based features. A candidate oscillation is classified as locomotor oscillation, when its normalized prominence-based feature *x*_*fea*_ exceeds the threshold τ_*fea*_:


$$p=\{\begin{array}{cc} 1,\; & \text{ if }x_{\text{fea}}>\tau_{\text{fea}}\\ 0, & \text{otherwise} \end{array}$$ (4)

In Equation 4, *x*_*fea*_ is one of the 12 prominence-based features, and *p* is the prediction result. To train for the optimal threshold, τ_*fea*_ goes through a parameter scanning from min(*x*_fea_) to *max*(*x*_fea_), and its optimal value is determined as the value associated with the highest F-1 score.

Approach B: Support Vector Machine (SVM) on multi-features. Additional features can often improve the accuracy of a classifier. Therefore, a Gaussian Radial Basis Function (RBF) kernel support vector machine (SVM) (Chang et al., 2010) is proposed to incorporate additional peak-features from Supplementary Appendix 3. This approach is beneficial as the RBF kernel’s capability to handle non-linear classification and map the features to a high-dimensional space where a linear separation is possible (MacQueen, 1967). A candidate is classified as locomotor oscillation when the feature-mapping of **X** ∈ ℝ^*N*_*fea*_^ (*N*_*fea*_ is the number of features) yields a positive result, as Equations 5, 6 shown:


$$p\;=\;\left\{ \begin{array}{l} 1,\text{ if }\sum_{i}\alpha_{i}c_{i}K\left(x_{i},x\right)+b\;>\;0\\ 0,\text{ otherwise } \end{array}\right.$$ (5)

$$K\,\left({\text{x}}_{i},{\text{x}}^{{}^{\prime }}\right)=\text{exp}\,\left(-\gamma \,{||{\text{x}}_{i}-{\text{x}}^{{}^{\prime }}||}^{2}\right)$$ (6)

Where *K* denotes the RBF kernel and *p* is the prediction result in Equation 6. The feature vector *x* encompasses a subset of normalized peak-features. The parameter *x_i_* denotes the support vectors; *c_i_* denotes the class of each support vectors; α_*i*_ denotes Lagrange multipliers associated with the support vectors; b denotes the bias term; and γ determines the shape of the kernel. All these parameters are learned during the training process.

Approach C: K-means clustering on multi-features. K-means was selected for its simplicity and efficiency, serving as an unsupervised baseline to evaluate the intrinsic cluster structure of the feature set without relying on labeled information, as compared to supervised learning of rbf-SVM (MacQueen, 1967). A candidate is classified as locomotor oscillation, when the distance between its feature *x* ∈ ℝ^*N*_*fea*_^ and the centroids of locomotor oscillation μ_1_ ∈ *R*^*N*_*fea*_^ is smaller than the distance between *x* and the centroids of spontaneous oscillation μ_0_ ∈ *R*^*N*_*fea*_^:


$$p=\left\{ \begin{array}{l} 1,\;if\;{\left|\left|x-\mu_{1}\right|\right|}^{2}<\;{\left|\left|x-\mu_{0}\right|\right|}^{2}\\ 0,\text{ otherwise} \end{array}\right.\;$$ (7)

In Equation 7, the feature vector **x** encompasses a subset of normalized peak-features; *p* is the prediction result. The parameters μ_**0**_ and μ_**1**_ are learned during the training process, and the cluster with a higher averaged prominence is assigned the True (locomotor oscillation) class.

### 2.6 Characterization of locomotor rhythm

Beyond identifying all the locomotor oscillations within CPG signals, the final step is to characterize the locomotor rhythm quantitatively based on the oscillation prediction results. In general, a successfully activated CPG response with a complete “depolarization-repolarization” process typically contained one locomotor episode. To find the episode, locomotor oscillations that were closely spaced (<2. 5= 1/0.15*Hz*) were merged. In certain instances, locomotor rhythm may contain multiple episodes spaced further apart due to external intervention to the CPG. To facilitate the analysis of locomotor rhythms, *k* rhythm-features are calculated for each channel (*k* = 12 in this study), as detailed in Supplementary Appendix 4. Five key features were frequently employed for CPG analysis: number of locomotor oscillation (“Num”), duration of locomotor rhythm (“Dur”), mean period of locomotor oscillation (“mPer”), coefficient of variation of locomotor oscillation (“PerCV”) and signal depolarization (“Dep”). The remaining features provide additional insights for a more comprehensive characterization.

## 3 Experiments

### 3.1 Dataset

In accordance with the guidelines of the National Institutes of Health (NIH) and with the Italian Animal Welfare Act 24/3/2014 n. 26, implementing the European Union directive on animal research (2010/63/EU), experiments were performed on 59 preparations of the entire central nervous system isolated from neonatal rats (0–2 days old), as previously reported (Mohammadshirazi et al., 2023). The animal protocol was approved by the Italian Ministry of Health with the notification. 22DAB.N.52M dated Oct 30th, 2019 and approved by SISSA ACUC (OPBA) committee (verbale n.17/3019). The whole dataset used in this study consisted of three independent subsets, which were collected under different experimental protocols, here indicated as A, B and C. In protocol A, the sacra-caudal afferents were stimulated (intensity = 37.5–160 μA, pulse duration = 0.1 ms, frequency = 2 Hz). To electrically induce fictive locomotion activities in protocol B, trains of rectangular pulses (intensity = 7.5–40 μA, pulse duration = 0.1 ms, frequency = 2 Hz) were applied for 80 s to either right lumbar 6 (L6) or right sacral 1 (S1) dorsal roots. Finally, in protocol C, a punctiform stimulation of the ventro-lateral medulla of the brainstem (intensity = 500–4,500 μA, pulse duration = 1–5 ms, frequency = 1–2 Hz) was applied for 80 s.

Extracellular recordings were performed in DC mode using tightly fitting monopolar suction electrodes from left and right lumbar 2 (L2) VRs, and left and right lumbar 5 (R5) VRs, corresponding to flexor and extensor related commands, respectively (Taccola et al., 2008).

Table 1 presents detailed information on the recording signals for each protocol. Although the sampling varied across protocols, it was uniformly down sampled to 500 Hz during the pre-processing step. Two expert experimentalists independently identified a signal as an oscillatory event based on its distinct deviation from the baseline and clear rhythmic characteristics. A signal was further attributed to locomotor activity (True class) when such oscillatory patterns alternated with subsequent signals from the homolateral root and exhibited coordinated alternation with corresponding ipsilateral L2 or L5 signals. In summary, the recording length across the entire dataset ranged from 79 to 120 s (on average:116.1 ± 10.7s). The oscillation count per channel varied from 59 to 154 (on average: 103.3 ± 15.6). The ratio of locomotor oscillation to non-locomotor oscillation ranged from 0 to 1.1 (on average: 0.37 ± 0.19).

**TABLE 1:** Dataset summary.

Protocol	A	B	C
Recording count	41	14	4
Osc count	13,076	5,123	1,732
TRUE	3,038	1,477	413
FALSE	10,038	3,646	1,319
Ratio (T/F)	1/3	2/5	1/3

### 3.2 Experiment design

To enhance the development and evaluation of the proposed algorithms, two experiments were conducted. Experiment 1 focused on feature selection and the initial evaluation of the model, utilizing data from protocol A. Experiment 2 aimed to assess the model’s generalizability, using data from all three protocols.

#### 3.2.1 Experiment 1

**FIGURE 3:**
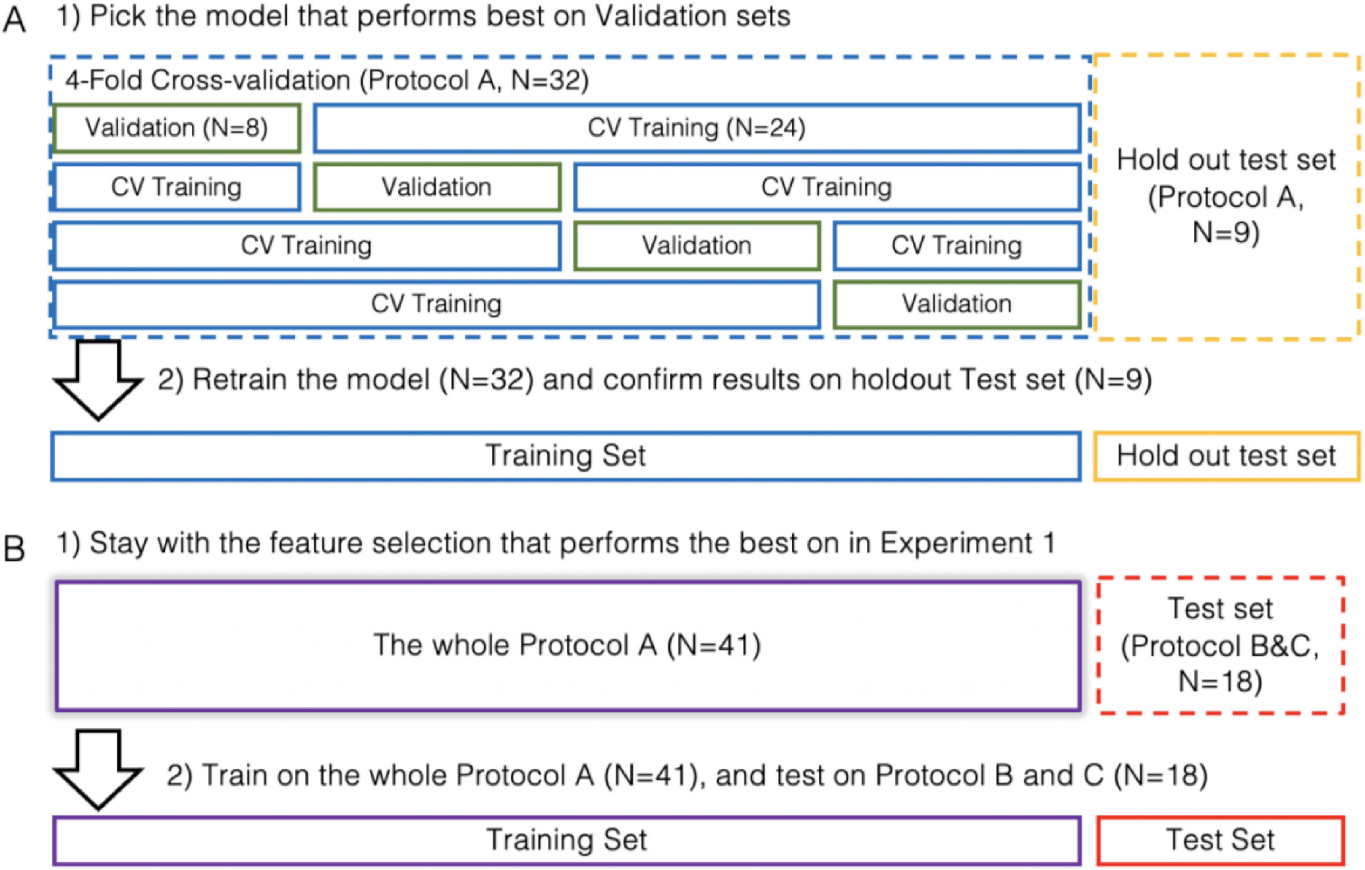
Experiment design. **(A)** Experiment 1 comprises of a cross-validation session and a test session. **(B)** Experiment 2 comprises of a training session and a test session.

Experiment 1 comprised of a four-fold cross-validation (CV) session and a test session (Figure 3A).

To prepare for the CV session, 9 out of 41 recordings of protocol A were randomly held out for the test session, while the remaining 32 recordings were evenly distributed into four groups. During the CV session, each group (*N* = 8) took turns serving as the validation set, while the remaining three groups (*N* = 24) served as the training set. For Approach A, 12 thresholding models were evaluated, each using one of the proposed peak features. For Approach B, a total of 1,024 SVM models were tested, each using a different subset of 12 peak features as input (detailed in Supplementary Appendix 3). The model with the highest validation performance—measured by the average F1 score across four folds—was selected as the optimal model for both Approach A and B. In the subsequent test session, the optimal thresholding model and the optimal SVM model were re-trained from scratch using the selected feature set and all 32 training recordings, and then evaluated on the held-out test set (*N* = 9). Lastly, for comparison, the K-means model (Approach C) was evaluated using the same feature set as the optimal SVM model in both the CV and test sessions.

#### 3.2.2 Experiment 2

Experiment 2 consisted of a training session and a test session (Figure 3B). During the training session, the optimal thresholding model and SVM model identified in Experiment 1 were re-trained from scratch using the selected feature set and all recordings from protocol A (*N* = 41). In the subsequent test session, the re-trained models were evaluated on recordings from protocols B and C (*N* = 18).

### 3.3 Evaluation

#### 3.3.1 Feature analysis

To assess the discriminative power of the proposed peak-features for distinguishing between the locomotor oscillation and non-locomotor oscillation, the entire dataset (all three protocols) was pooled together to visualize the distribution of each feature. Next, statistical tests were conducted to compare the mean differences between the two classes of oscillations for each feature. For binary features, including “FreqCK,” “StimCK” and “nSynCK,” the Chi-square test was applied, otherwise the Student’s *t*-test was applied (Pearson, 1895, 1992). Additionally, a correlation matrix was computed to assess the relationship among all peak-features and the oscillation ground-truth labels. When two variables are both binary, the Spearman correlation coefficient was applied, otherwise Pearson correlation was applied *(De Winter et al., 2016)*.

#### 3.3.2 Oscillation classification

To comprehensively evaluate the overall classification performance of proposed algorithms, four metrics were employed for both Experiment 1 and Experiment 2. The four metrics included: sensitivity (SENS), precision (PREC), accuracy (ACC) and F-1 score (F1). The metrics were defined as Equations 8–11, shown as follows:


$$S\,E\,N=\frac{T\,P}{T\,P+F\,N}$$ (8)

$$P\,R\,E=\frac{T\,P}{T\,P+F\,P}$$ (9)

$$A\,C\,C=\frac{T\,P+T\,N}{T\,P+T\,N+F\,P+F\,N}$$ (10)

$$F\,1=\frac{2\times S\,E\,N\times P\,R\,E}{S\,E\,N+P\,R\,E}=\frac{2\,T\,P}{2\,T\,P+F\,P+F\,N}$$ (11)

where TP, FP, TN, FN referred to true positive, false positive, true negative and false negative, respectively, which were counted by comparing prediction and ground truth label. The F-1 score, calculated as the harmonic mean of precision and sensitivity, played a critical role in addressing the imbalanced True/False class issue in the dataset. Therefore, it served as the criterion for feature selection during cross-validation session (Taha and Hanbury, 2015).

#### 3.3.3 Locomotor rhythm characterization

The model trained in Experiment 2 was further deployed on the test set to calculate the 12 rhythm-features using the classification results given by each approach. Human labels were also utilized to calculate the rhythm-features and served as ground truth. To evaluate the results, intraclass correlation coefficient (ICC) was applied between calculations given by the proposed approach and human labels, where a higher value of ICC indicated a higher agreement, and thus a more reliable approach for automatic analysis (Bartko, 1966).

## 4 Results

### 4.1 Feature analysis

#### 4.1.1 Discriminative power

**FIGURE 4:**
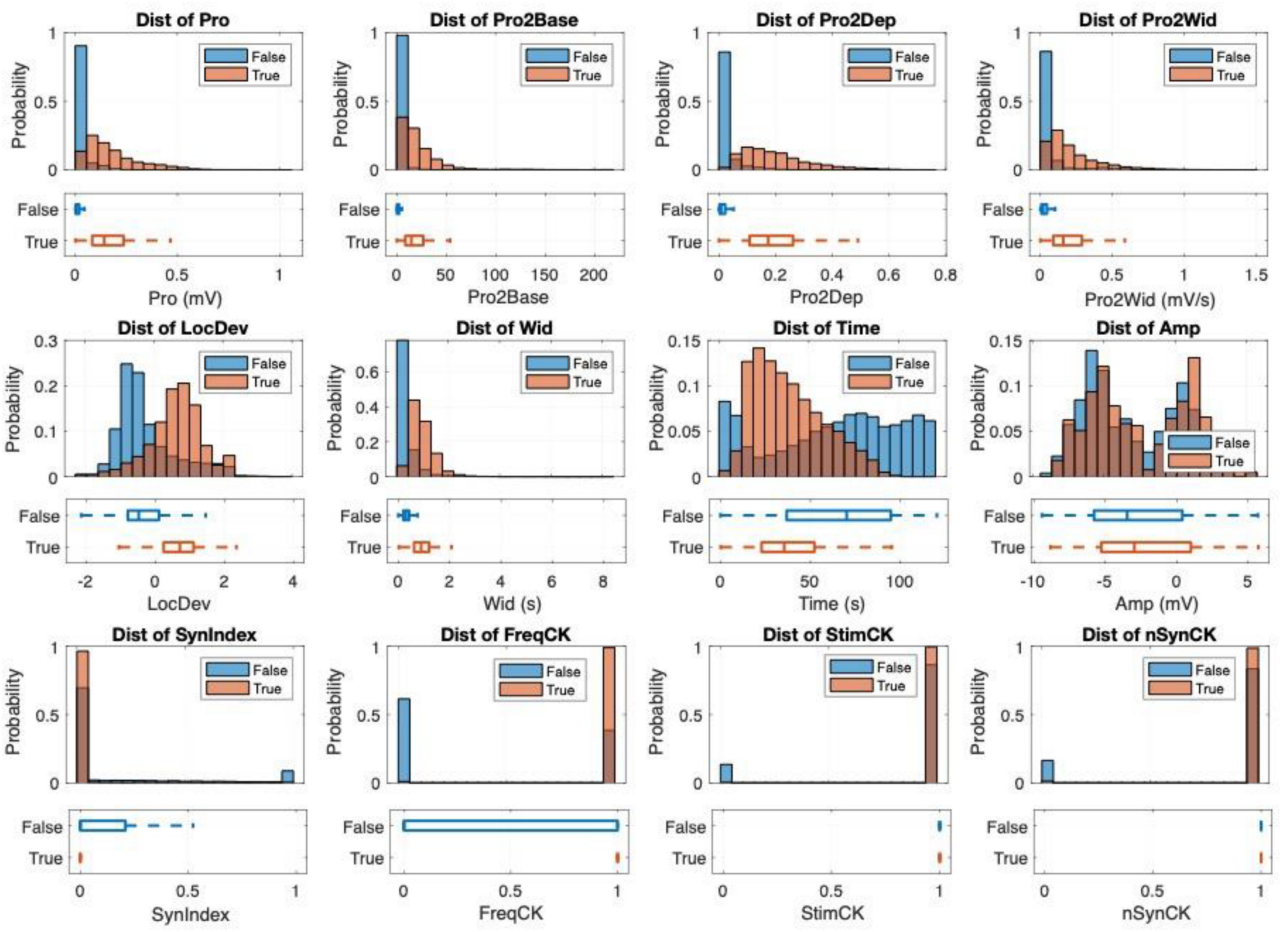
Peak-features analysis: the distribution of features, with True class (locomotor oscillation) and False class (non-locomotor oscillation) plotted separately.

The discriminative power of each of 12 peak-features was analyzed by examining the distributions of their output values for each ground truth class (True, locomotor oscillation vs. False, non-locomotor oscillation), which are summarized in Figure 4. The resulting observations were:

1. Prominence-related features (“Pro,” “Pro2base,” “Pro2Dep,” “Pro2Wid,” and “LocDev”) and “Wid” feature were usually higher for locomotor oscillations than for non-locomotor oscillation.
2. “Time” feature for locomotor oscillations distributed less in the beginning and the end of recording, likely because in the first few seconds, the stimulation has not yet started to activate locomotion, while toward the end of recording, locomotion gradually diminished and stopped.
3. “Amp” (signal amplitude) feature revealed minimal differences between classes because the signal baseline level varied significantly among recordings and channels.
4. “SynIndex” feature showed zero synchronicity-degree for most oscillation cycles of both classes. But the locomotor oscillations demonstrated a significantly higher frequency of zero synchronicity, because locomotor oscillations typically have phase difference across channels due to the natural flexor-extensor coordination and the left-right gait alternation.
5. “FreqCK” and “StimCK” features were mostly equal to True for locomotor oscillations, implying that their frequency adhered to the locomotion bandwidth and rarely occurred in the absence of stimulation.

Finally, all 12 features demonstrated a good representation of physical characteristics of CPG oscillations and yield a remarkably small *p*-value ( < 10^−6^) in the statistical tests of mean differences, implying their good potential in discriminate between two classes of oscillation.

#### 4.1.2 Correlation analysis

**FIGURE 5:**
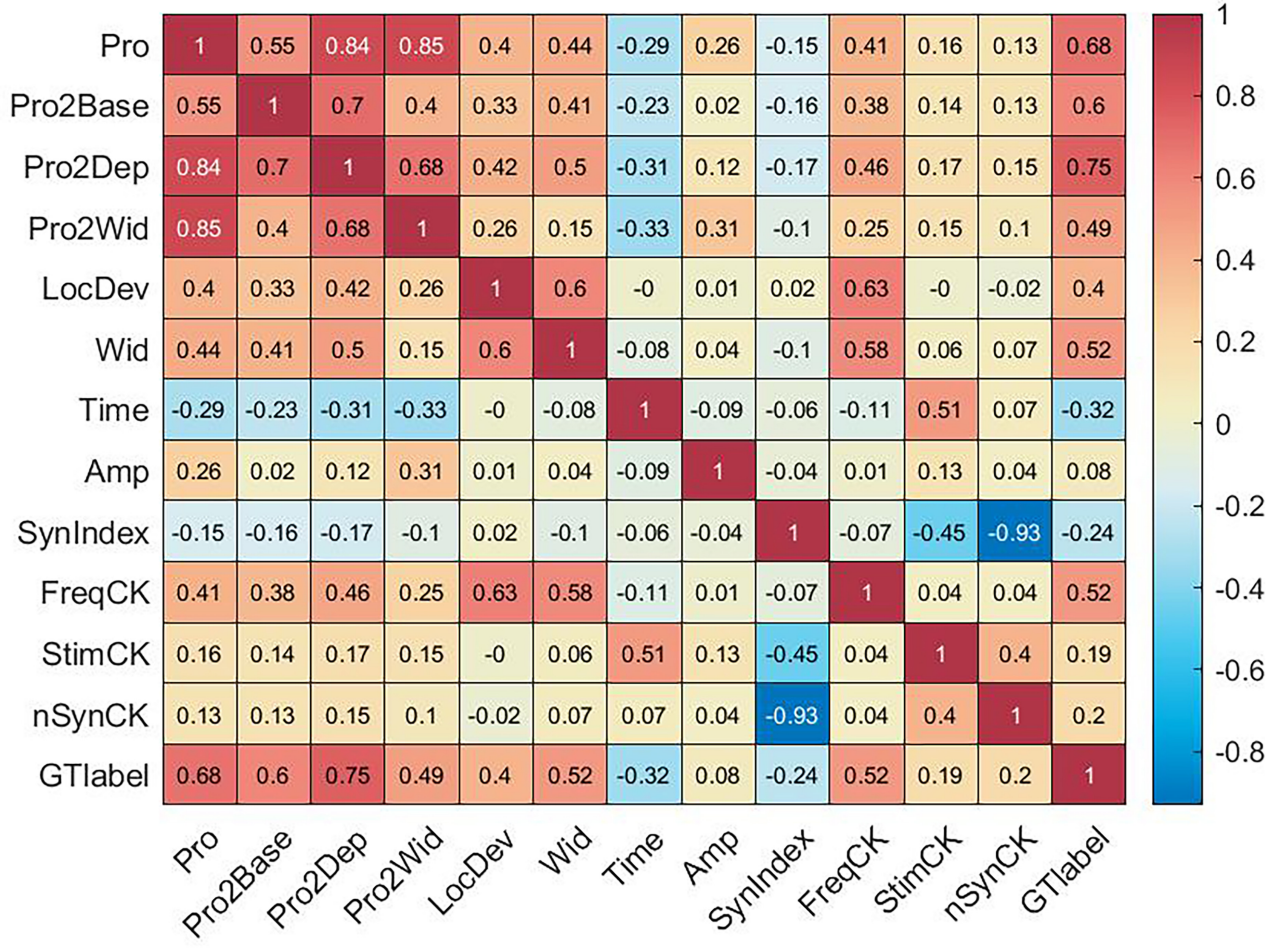
Peak-features analysis: correlation matrix among features and ground truth (GT) label.

To further assess the relationship among all classification features and oscillation classes (ground truth label), a thorough correlation analysis was conducted by computing their correlation matrix (Figure 5). The top three most correlated features were: “Pro2Dep” (0.75), “Pro” (0.68), “Pro2Base” (0.6), suggesting their strong potential for classification. Notably, some features had high correlation with each other:

1. Prominence related features and “Wid” exhibited very high correlation due to their interdependent definitions.
2. Strong correlations were observed between in the paired features of “Wid” and “FreqCK,” “Time” and “StimCK,” “SynIndex,” and “nSynCK” since each Boolean feature was the dichotomization of another feature from the pair.
3. “Amp” was positively correlated with prominence related features, while “Time” was negatively correlated with prominence related features, suggesting that oscillations with higher prominence often occurred earlier in time and manifested with greater amplitude (riding on depolarization).

### 4.2 Locomotor-oscillation detection

#### 4.2.1 Experiment 1

**FIGURE 6:**
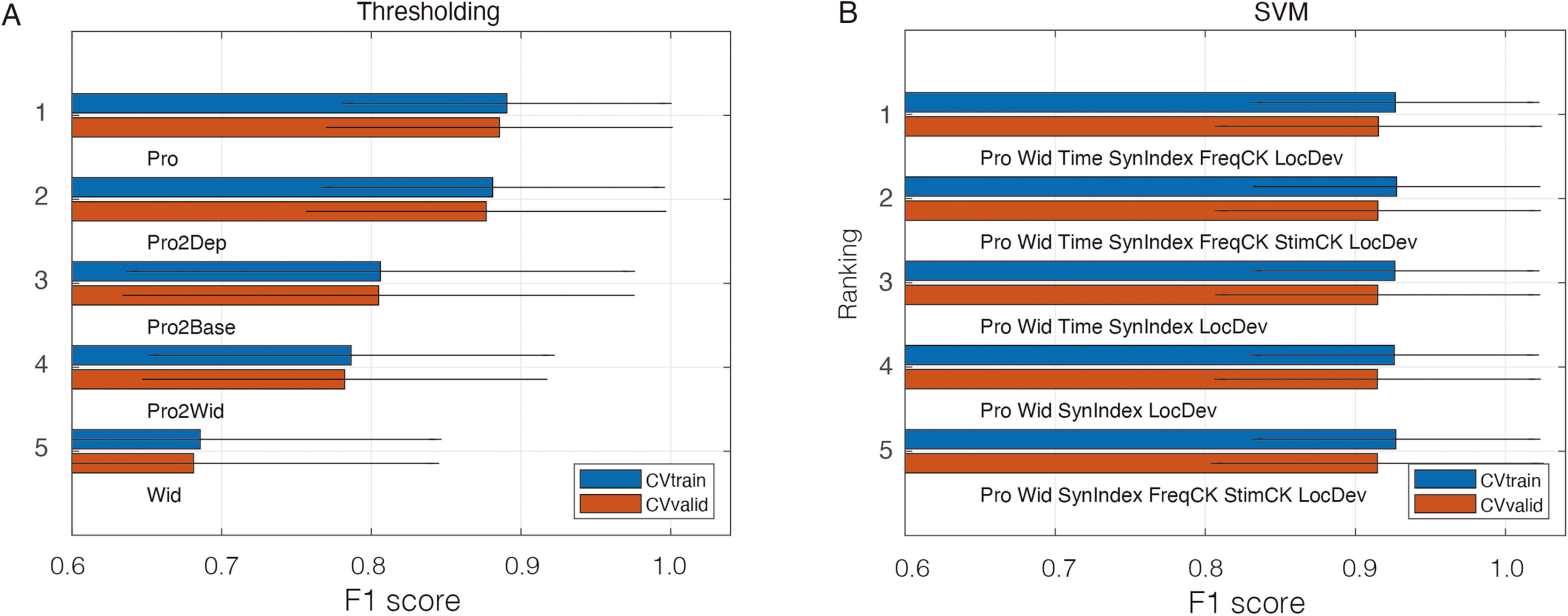
**(A)** The top performance of thresholding approach. **(B)** The top performance of SVM approach.

Experiment 1 consisted of a CV session and a test session, using data from Protocol A. In the CV session, one objective was to select the optimal feature-set for classification. We tested 12 proposed peak features for thresholding approach and tested a total of 1,024 feature-sets for SVM approach. Figure 6 listed the top performed models for each approach. For thresholding models, “Pro” feature exhibited a F-1 score of 0.886 ± 0.112, representing the highest performance on CV validation sets. This suggested the proposed “prominence” feature was able to discriminate locomotor oscillations from non-locomotor oscillations (McCrea, 2001). Notably, normalization was applied to the feature within each channel prior to any computation. For SVM models, the feature set (Pro, Wid, Time, SynIndex, FreqCK, LocDev) scored a F-1 score of 0.915 ± 0.105, representing the highest performance on CV validation sets. This indicated that the inclusion of features alongside prominence—such as oscillation width, timestamp, synchronicity index, frequency check and local deviation–improved the performance.

The comprehensive performances of Experiment 1 are listed in Table 2. It was evident that SVM approach consistently achieving an average score of over 0.9 for almost every metric in both CV session and test session. In comparison, the thresholding approach, while yielding good results, fell short of the SVM’s performance. Both approaches benefited from the proposed peak-based feature extraction framework, underscoring its robustness and reliability. Although K-means used the same feature set as the SVM, its lower performance which resulted from the absence of label guidance highlights the advantages of supervised learning in the SVM approach. Nevertheless, with an F1 score above 0.774 and an accuracy of 0.890, K-means still demonstrated the strong clustering structure inherent in the SVM-selected feature set.

**TABLE 2:** Performance summary of Experiment 1.

Metric	Approach	Cross-validation		Test	
		Training	Validation	Re-training	Test
Precision	Thresholding	0.877 ± 0.154	0.876 ± 0.160	0.879 ± 0.155	0.866 ± 0.190
	SVM	**0.931 ± 0.126**	**0.925 ± 0.138**	**0.931 ± 0.128**	**0.912 ± 0.158**
	Kmeans	0.671 ± 0.179	0.671 ± 0.179	0.671 ± 0.179	0.688 ± 0.176
Sensitivity	Thresholding	0.928 ± 0.075	0.921 ± 0.081	0.923 ± 0.077	0.886 ± 0.078
	SVM	0.938 ± 0.076	0.922 ± 0.084	0.938 ± 0.079	0.900 ± 0.073
	Kmeans	**0.942 ± 0.063**	**0.942 ± 0.063**	**0.942 ± 0.063**	**0.910 ± 0.102**
Accuracy	Thresholding	0.952 ± 0.036	0.950 ± 0.039	0.952 ± 0.037	0.942 ± 0.040
	SVM	**0.970 ± 0.028**	**0.965 ± 0.033**	**0.969 ± 0.030**	**0.958 ± 0.035**
	Kmeans	0.876 ± 0.065	0.876 ± 0.065	0.876 ± 0.065	0.890 ± 0.051
F-l	Thresholding	0.890 ± 0.106	0.886 ± 0.112	0.889 ± 0.107	0.861 ± 0.122
	SVM	**0.927 ± 0.092**	**0.915 ± 0.105**	**0.926 ± 0.095**	**0.898 ± 0.106**
	Kmeans	0.768 ± 0.137	0.768 ± 0.138	0.768 ± 0.138	0.774 ± 0.147

Bold values indicate the best performance within each metric and dataset.

#### 4.2.2 Experiment 2

Experiment 2 consisted of a retraining session and a test session, where the model was trained on data from protocol A and tested on data from protocol B and C. The comprehensive performances are listed in Table 3. Similarly, to Experiment 1, SVM approach-maintained superiority over others, and K-means trailed as the least effective.

**TABLE 3:** Performance summary of Experiment 2.

Metric	Approach	Training	Test		
			Overall	Protocol B	Protocol C
Precision	Thresholding	0.856 ± 0.171	0.916 ± 0.146	0.916 ± 0.098	0.915 ± 0.252
	SVM	**0.921 ± 0.138**	**0.947 ± 0.135**	**0.953 ± 0.075**	0.928 ± 0.25
	Kmeans	0.675 ± 0.178	0.763 ± 0.216	0.712 ± 0.179	**0.933 ± 0.249**
Sensitivity	Thresholding	**0.940 ± 0.061**	0.929 ± 0.073	0.916 ± 0.076	0.977 ± 0.029
	SVM	0.933 ± 0.075	0.919 ± 0.080	0.921 ± 0.081	0.912 ± 0.080
	Kmeans	0.935 ± 0.074	**0.947 ± 0.124**	**0.939 ± 0.139**	**0.977 ± 0.029**
Accuracy	Thresholding	0.951 ± 0.039	0.957 ± 0.04	0.952 ± 0.029	0.972 ± 0.063
	SVM	**0.967 ± 0.031**	**0.965 ± 0.034**	**0.965 ± 0.031**	0.968 ± 0.042
	Kmeans	0.879 ± 0.062	0.896 ± 0.095	0.872 ± 0.09	**0.976 ± 0.064**
F-l	Thresholding	0.884 ± 0.119	0.911 ± 0.129	0.911 ± 0.066	0.914 ± 0.246
	SVM	**0.919 ± 0.099**	**0.923 ± 0.127**	**0.933 ± 0.062**	0.888 ± 0.242
	Kmeans	0.770 ± 0.139	0.824 ± 0.195	0.795 ± 0.169	**0.924 ± 0.247**

Bold values indicate the best performance within each metric and dataset.

Additionally, each classification approach showed different performance between protocols. While thresholding and K-means approach performed better on Protocol C as compared to Protocol B, SVM approach performed better on Protocol B than Protocol C.

**FIGURE 7:**
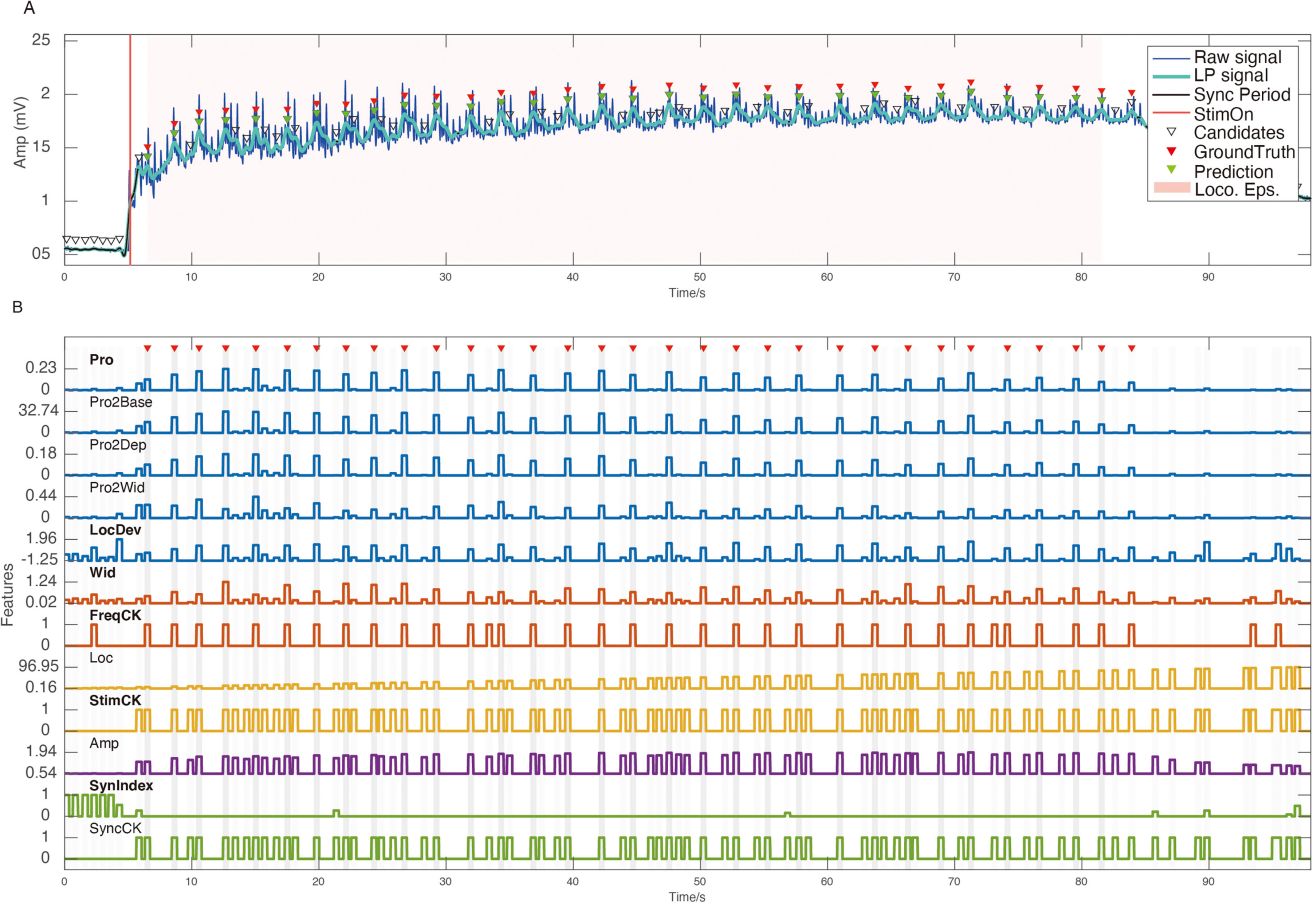
Locomotor oscillation detection example (flexor-related lumbar ventral root, VRlL2). **(A)** Temporal waveform of the CPG signal. **(B)** Visualization of values of 12 peak-features corresponding to each candidate oscillation in **(A)**.

Figure 7 illustrates an example of detecting a locomotor oscillation using SVM approach from Experiment 2, where the values of peak-features in panel B are aligned with the timestamp of their corresponding oscillation peaks in panel A.

#### 4.2.3 Performance comparison among experiment sets

**FIGURE 8:**
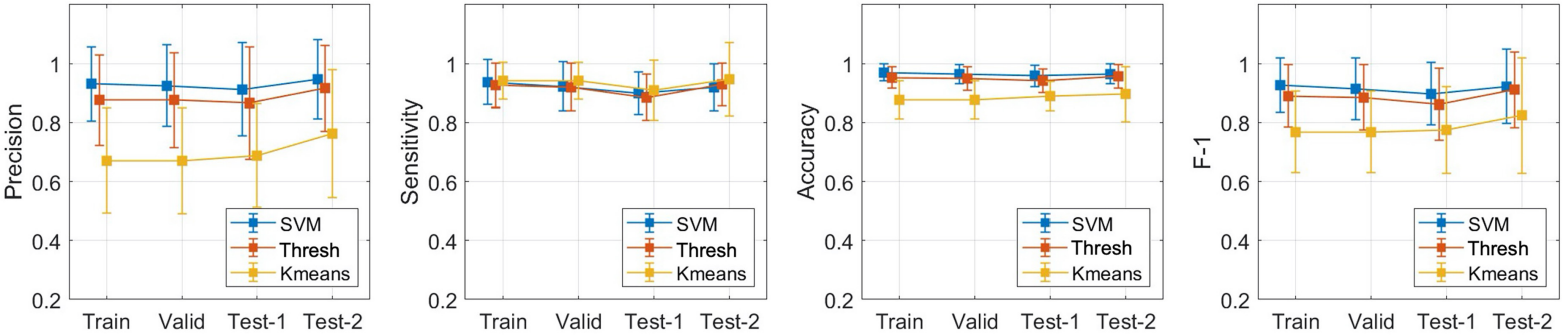
Detection performance comparison among four experiment sets.

To clearly illustrate the difference among experimental sets, Figure 8 pooled results from four sets across two experiments together, including the CV training performance, CV validation performance and test performance from Experiments 1 and 2. It was evident that, for thresholding and SVM, both accuracy and F-1 score showed the same pattern, with performance in CV training (thresholding: ACC = 0.952 ± 0.036, F-1 = 0.890 ± 0.106; SVM: ACC = 0.970 ± 0.028, F-1 = 0.927 ± 0.092) surpassing that in CV validation (thresholding: ACC = 0.950 ± 0.039, F-1 = 0.886 ± 0.112; SVM: ACC = 0.965 ± 0.033, F-1 = 0.915 ± 0.105), and followed by test 1 (thresholding: ACC = 0.942 ± 0.040, F-1 = 0.861 ± 0.122; SVM: ACC = 0.958 ± 0.035, F-1 = 0.898 ± 0.106). This phenomenon is commonly observed in supervised models because these models are trained to fit the training data accurately but may struggle in generalizing to unseen data. In contrast, this issue was not evident in an unsupervised approach, such as K-means.

Notably, an independent test set 2 (thresholding: ACC = 0.957 ± 0.040, F-1 = 0.911 ± 0.129; SVM: ACC = 0.965 ± 0.034, F-1 = 0.923 ± 0.127) performed as good as test set 1, substantiating the great generalizability of the proposed models.

### 4.3 Locomotor rhythm characterization

The locomotor rhythm is characterized by 12 rhythm-features using the classification results from each approach and the ground truth label (Table 4). The SVM-based calculations showed the closest results with the ground truth (GT), achieving the highest ICC, which indicates strong alignment with human expert assessments.

**TABLE 4:** Summary of rhythm features calculations a approach and GT (higher ICC was marked as bold).

Approach	SVM			Thresholding			Kmeans			GT	
	Mean	Std	ICC	Mean	Std	ICC	Mean	Std	ICC	Mean	Std
Num	25.76	6.59	**0.81**	26.53	6.09	0.69	32.44	9.42	0.32	27.00	8.14
mPer	2.39	0.88	0.88	2.42	0.86	**0.90**	2.61	0.86	0.84	2.37	0.79
PerCV	0.23	0.09	**0.40**	0.26	0.10	0.34	0.28	0.11	0.36	0.21	0.09
Dur	54.86	15.69	**0.81**	56.85	15.72	0.75	73.33	8.95	0.00	59.77	17.08
Dep	1.01	0.66	**0.97**	0.99	0.66	0.96	0.94	0.59	0.83	1.02	0.70
AUC	50.14	28.65	**0.92**	51.92	28.71	0.89	68.00	40.88	0.69	55.42	31.87
Duty	0.44	0.12	**0.94**	0.44	0.12	0.93	0.42	0.12	0.84	0.44	0.12
mWid	1.01	0.36	**0.96**	1.01	0.36	0.96	1.06	0.34	0.91	1.00	0.35
WidCV	0.27	0.13	**0.66**	0.32	0.18	0.50	0.37	0.18	0.41	0.28	0.13
mPro	0.27	0.11	**0.98**	0.27	0.11	0.98	0.23	0.10	0.86	0.26	0.10
ProCV	0.28	0.12	**0.80**	0.28	0.13	0.74	0.43	0.23	0.50	0.31	0.12
mLocDev	0.82	0.55	**0.92**	0.88	0.53	0.91	0.99	0.49	0.85	0.81	0.56

Following the guidelines (Koo and Li, 2016), the ICC of key locomotor rhythm features were reported:

1. For number of oscillations (“Num”), there was good agreement between SVM and GT (ICC = 0.81), while the agreement between thresholding and GT was also considered moderate (ICC = 0.69).
2. For mean period of oscillation (“mPer”), all three approaches had good agreement with GT (ICC > 0.8).
3. For coefficient of variation of period of locomotor oscillation (“PerCV”), SVM approach had an agreement with GT (ICC = 0.4), outperforming other approaches.
4. For duration of oscillation (“Dur”), both SVM and thresholding had good agreement with GT (ICC > 0.75).
5. For Depolarization (“Dep”), all three approaches had good agreement with GT (ICC > 0.8).

In summary, SVM proved to be reliable in calculating all 12 features in a good agreement with GT. Specifically, six features showed excellent agreement (“Dep,” “Duty,” “mWid,” “AUC,” “mPro” and “mLovDev” with ICC > 0.9), and four showed good agreement (“Num,” “mPer,” “Dur,” “ProCV” with 0.9 > = = ICC > 0.75). Notably, 11 out of the 12 rhythm-features calculated by SVM ranked highest among the three approaches. As the SVM performed best in both oscillation classification and locomotor rhythm characterization, it underscores the importance of precise detection and classification of locomotor oscillations for accurately characterizing locomotor rhythms.

## 5 Discussion

### 5.1 Analyzing the variance of dataset’s performance

In machine learning, an important objective is to develop models with strong generalizability. These models are typically trained on historical data but are intended to make predictions on unseen and more diverse datasets (L’Heureux et al., 2017). Variability among datasets arises from the difference in experimental protocols, subjects, devices or environments (Park et al., 2018). Therefore, assessing the robustness of models during their development becomes crucial.

In this study, we employed a standard machine-learning experimental design to evaluate both the effectiveness of various proposed models and their generalizability using data from multiple independent experiments with different protocols. As shown in Tables 2, 3, the performance across three protocols was deemed satisfactory, with the lowest F-1 score of 0.888 ± 0.242 still considered good. This success is attributed to the proposed peak-based framework, which introduced accurate, representative, and robust features for each oscillation. Additionally, the prosed peak-based feature extraction framework plus feature selection process effectively mitigated the risk of overfitting.

However, performance variations among protocols were evident. As shown in experiment 2, classifiers trained on Protocol A, performed significantly different on data from Protocol C compared to Protocol B. This result was presumed to arise from the differences between the training set and test set. Specifically, when the features distribution of training set and test set were more similar, the performance gap tended to be smaller. To investigate the potential relationship between data similarity and performance, we applied Kullback-Leibler (KL) divergence (Csiszar, 1975) to measure the similarity between two datasets in their respective feature space, as Equation 12 shown:


$$KL\left(P||Q\right)=\sum P\left(x\right)\text{log}\left(\frac{P\,\left(x\right)}{Q\,\left(x\right)}\right)$$ (12)

where *P*(*x*) and *Q*(*x*) represent the probabilities of observing the value x in feature distributions P and Q, respectively. The closer the value of KL divergence to 0, the greater the similarity between the two distributions. Table 5 lists the KL divergence of the features between the training set and the two test set protocols. The feature “Pro” exhibits higher similarity between the training set and Protocol C compared to Protocol B. This can explain why the thresholding model, which relies solely on the feature “Pro,” performed better on Protocol C than the Protocol B in Experiment 2 (scoring F-1prot.C: 0.914 ± 0.246 F-1prot.B: 0.911 ± 0.066, respectively).

**TABLE 5:** K-L divergence between training and test set data (lower divergence was marked as bold).

Protocol	Prominence	Width	Timestamp	Synchronicity	Freq check	Local Dev	Ave
Training-test pro.B	5.27E-07	5.27E-07	**1.91E-03**	**6.62E-02**	**4.31E-03**	**2.93E-04**	**1.21E-02**
Training-test pro.C	**2.25E-07**	**2.25E-07**	9.54E-01	1.99E-01	2.61E-01	1.68E-02	2.38E-01

When additional peak-features were included, the averaged KL values indicated that Protocol C was notably more distinct from the training set compared to Protocol B. This observation may explain why the SVM classifier, which utilizes multiple peak-features, performed significantly better on Protocol B (F-1prot.B: 0.933 ± 0.062 F-1prot.C: 0.888 ± 0.242). Although K-L divergence may not explain all performance differences, it served as a valuable tool for predicting the model’s outcome on new and unseen data, offering useful insight for model training and selection. By quantifying the distributional similarity between datasets, it enables an informed assessment of transferability, helping to prioritize training models on data that more closely match the target domain, making it particularly beneficial in data-scarce or resource-limited scenarios.

### 5.2 Training-size analysis

In practice, annotated data is both valuable and costly to obtain, making it essential to estimate the minimum training size needed for satisfactory performance. To explore this, we extended Experiment 1 by progressively increasing the training set size and monitoring corresponding changes in model performance. To achieve this, data under Protocol A (*N* = 41) was randomly divided to five groups of eight or nine recordings. In five experimental rounds, each group was used once as test set 1, while the remaining four formed the training pool. For each round, training sets of varying sizes (1–30) were randomly drawn from the pool, and the model was trained and evaluated on test set 1, followed by evaluation on an independent test set 2 (Protocol B and C, *N* = 18). To reduce selection bias, the process was repeated 10 times per training size.

**FIGURE 9:**
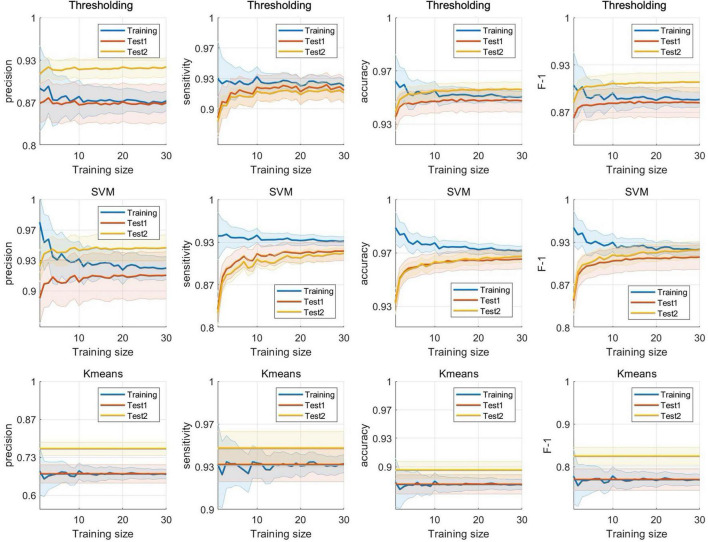
Training size test. Each subplot showed the results of extended experiment for different models and evaluation metrics.

Figure 9 shows plots of the extended experiment which displays a consistent trend across all four metrics for both thresholding and SVM approach. As training size increased, training performance decreased, exhibiting smaller standard errors, while the performance on both test set 1 and 2 increased. This trend reflects the model’s initial overfitting to small training sets. With larger training sizes, the model becomes less overfit, resulting in slightly lower training accuracy but improved generalization to unseen test data (Ying, 2019). It appears that the test performance begins to stabilize around a training size of 10, where satisfactory performance is achieved with a relatively small amount of annotated data for this dataset, although variability remains across metrics and repetitions. This result may serve as a reference for dataset sample size selection, but remains highly dependent on variability across different datasets. In contrast, the unsupervised K-means model exhibited flat trends for all metrics, as its performance was not affected by training size.

In summary, for reliable performance when applying POCA to a new, unseen dataset, it is recommended to use a model trained on data with similar signal characteristics. If such a model is unavailable, annotating a small number of oscillations from the new dataset and retraining POCA using the selected peak features can still yield satisfactory classification accuracy with minimal labeling effort.

### 5.3 Epoch-based and Peak-based feature extraction framework for detecting biological oscillations

In this study, instead of inheriting the conventional epoch-based feature extraction framework, a novel peak-based framework was proposed, where features were extracted at the level of each oscillation peak rather than epochs. The choice between these frameworks depends on the specific task and the available dataset. For instance, when working with oscillations that are sensitive to peak morphology, such as electrically induced fictive locomotor oscillations, the peak-based framework is advantageous due to its ability to capture “local characteristics,” which allows it to discern peak shapes effectively. On the other hand, when the analysis prioritizes characterizing a burst of oscillations, such as in seizure recordings or other HFO activity, the epoch-based framework may also yield good results. This is because epoch-features can capture “regional characteristics,” such as an energy hill (Staba et al., 2002).

However, incorporating features that capture “regional characteristics” within the peak-based framework is possible and beneficial. For example, Figure 6 illustrates that including the “LocDev” feature, which assesses the regional characteristic of a peak’s prominence relative to neighboring peaks, improved the performance of the SVM model within the peak-based framework. It is the inclusiveness of the peak-based framework, which accommodates both local and regional characteristics, that enables it to achieve strong performance on such problems. By adopting such a framework, the proposed method improves generalizability while reducing user-dependent parameterization that is often required by more straightforward approaches, which tend to lack robustness when faced with variability across recording channels, experimental subjects, and protocols. Beyond CPG signal analysis, this peak-based framework offers a potential new approach for detecting biological oscillations, with potential applications in similar tasks and analyses, such as HFO detection, seizure detection using EEG, etc.

## 6 Conclusion

In this paper, we reported a novel peak-based feature extraction framework and implemented an automated algorithm to alleviate the burden of the laborious manual data analysis, a problem amplified by the current trend toward design of more flexible CPG stimulation protocols. Notably, the peak-based feature extraction framework concentrates on extracting features at the level of oscillation peak rather than epochs, capturing both local and regional characteristics of the activity. This design yields high accuracy and generalizability across datasets from multiple protocols. The proposed peak-based oscillation classification algorithm (POCA) stands as a reliable and objective automatic tool for CPG locomotor signal analysis. More importantly, it facilitates large-scale data analysis during the fine-tuning of biomimetic protocols through substantial experimental trials.

## Data Availability

The raw data supporting the conclusions of this article will be made available by the authors, without undue reservation.
