# Supplementary material for: POCA: a CPG signal analysis algorithm using peak-based feature extraction and machine learning
Source: Front Neurosci. 2026 Mar 11;20:1740554. doi: 10.3389/fnins.2026.1740554 (PMC13013536; doi:10.3389/fnins.2026.1740554)
Supplement: Supplementary file 1 [file Table_1.pdf]

POCA: A CPG Signal Analysis Algorithm Using *Peak-based* Feature Extraction and Machine Learning

Appendix

Appendix A: Abbreviations summary

POCA	Peak-based oscillation classification algorithm	NN	Neural network
CPG	Central pattern generator	EEG	Electroencephalogram
rbf-SVM	Radius basis kernel support vector machine	CV	Cross-validation
SCI	Spinal Cord Injury	SENS	Sensitivity
FL	Fictive locomotion	PREC	Precision
DR	Dorsal root	ACC	Accuracy
VR	Ventral root	ICC	Intraclass correlation coefficient
EMG	Electromyography	GT	Ground Truth
HFO	High frequency oscillation	KL	Kullback-Leibler
LDA	Linear discriminate analysis		

Appendix B: Algorithm pipeline

Peak-based oscillation classification algorithm (POCA) for CPG signal analysis

Input:

N_{chan} channels CPG signals $y^{(c)} \in \mathbb{R}^n$ $(c = 1, \dots, N_{chan})$

Output:

Peak-feature matrix $X^{(c)} \in \mathbb{R}^{\mathrm{N}_{\mathrm{osc}}^{(c)} \times m}$ (m peak-features, $\mathrm{N}_{\mathrm{osc}}^{(c)}$ oscillations)

Prediction vector $p^{(c)} \in \mathbb{B}^{N_{osc}^{(c)}}$ (locomotor: $p = True$; non locomotor: $p = False, \mathrm{N}_{\mathrm{osc}}^{(c)}$ oscillations)

Locomotor rhythm-features vector $z^{(c)} \in \mathbb{R}^k$ (k rhythm-features)

For *c*-th channel:

1: **Pre-processing:** Low pass filtering $y^{(c)}$ to get lowpass signal $y_{\mathrm{LP}}^{(c)}$

2: **Synchronicity calculation:** Calculate the synchronicity signal with Boolean values $s^{(c)}$, where synchronicity is True when its phase differences with the rest channels are less than 45°, otherwise synchronicity is False.

3: **Oscillations detection and feature extraction:** Detect all candidate oscillations by finding the local maxima points of $y_{\mathrm{LP}}^{(c)}$. Each oscillation is characterized by m peak-features based on information given by $y_{\mathrm{LP}}^{(c)}$ and $s^{(c)}$. This step outputted a feature matrix of detected oscillations: $X^{(c)} \in \mathbb{R}^{\mathrm{N}_{\mathrm{osc}}^{(c)} \times m}$.

4: **Oscillations classification:** Classify each candidate oscillation as either True (locomotor oscillation) or False (non-locomotor oscillation), the prediction labels $p^{(c)}$was given by $p^{(c)} =$

$f(X^{(c)})$ where f denotes the prediction function of one the following approaches: thresholding, Support Vector Machine, and K-means clustering

5: **Locomotor rhythm characterization:** The locomotor rhythm is characterized by k rhythm-features based on the features matrix of detected oscillations $X^{(c)}$ and their prediction vector $p^{(c)}$. This step outputted a rhythm-feature vector $z^{(c)} \in \mathbb{R}^k$.

Appendix C: Definition and calculation of proposed peak-features

Features name	Definition/calculation	Calculation
Prominence (Pro)	oscillation magnitude	$Pro = y_{\mathrm{LP}}^{(c)}(t) - \mathrm{Ref}$
Width (Wid)	oscillation width	$Wid = y_{\mathrm{LP}}^{(c)}(t_R) - y_{\mathrm{LP}}^{(c)}(t_L)$
Amplitude (Amp)	oscillation absolute amplitude	$Amp = y_{\mathrm{LP}}^{(c)}(t)$
Timestamp (Time)	oscillation timestamp	$Time = t$
Pro/Dep ratio (Pro2Dep)	oscillation prominence compared to the signal depolarization	$Pro2Dep = \frac{Pro}{\mathrm{Dep}}$
Pro/Base ratio (Pro2Base)	oscillation prominence compared to the mean prominence of baseline period oscillations	$\mathrm{Pro2Base} = \frac{Pro}{\mathrm{mean}(Pro_{baseline})}$
Pro/Wid ratio (Pro2Wid)	oscillation prominence compared to its width	$Pro2Wid = \frac{Pro}{Wid}$
Local deviation (LocDev)	oscillation prominence deviation from its temporal neighbors ($N_{nb} = 4$)	$LocDev = \frac{Pro - \mathrm{mean}(Pro_{nb})}{\mathrm{std}(Pro_{nb})}$
Synchronous Index (SynIndex)	degree of synchronicity within 0.4s window of synchronicity signal	$Sync = \frac{\sum_{[t-0.2,t+0.2]} s^{(c)}(t)}{0.4 * fs}$
Stimulation check (StimCK)	whether the oscillation happen after stimulation onset	$StimCK = \begin{cases} \mathrm{True},, t > t_{\mathrm{StimOnset}} \\ \mathrm{False}, \quad \mathrm{otherwise} \end{cases}$
Frequency check (FreqCK)	whether the oscillation is within the FL bandwidth	$FreqCK = \begin{cases} \mathrm{True}, \frac{1}{2 * Wid} \in [0.15,1.5]\mathrm{Hz} \\ \mathrm{False}, \quad \mathrm{otherwise} \end{cases}$
Non-sync check (nSynCK)	whether synchronicity within 0.4s window is smaller than 50%	$nSynCK = \begin{cases} \mathrm{True}, SynIndex < 50\% \\ \mathrm{False}, \quad \mathrm{otherwise} \end{cases}$

c: channel index; f_s: sampling frequency.
t: oscillation timestamp; t_R, t_L: left/right timestamp of the intercept of half prominence and the signal. $t_{\mathrm{Stimonset}}$: timestamp of stimulation onset.
$y_{\mathrm{LP}}^{(c)}$: low passed signal; $s^{(c)}$: synchronicity signal.
Ref: reference level to measure the prominence, as shown in Figure 2A; Dep: depolarization, as shown in Figure 2B.
$Pro_{baseline}$: Prominence of baseline period oscillations, as shown in Figure 2B; Pro_{nb}: Prominence of temporal neighors, as shown in Figure 2B.

Appendix D: Definition and calculation of locomotor rhythm-features

Features	Description	Calculation
Num	Number of oscillations within all locomotor episodes	$Num = \sum_i N_i$
Dur	Total duration of all locomotor episodes	$Dur = \sum_i (t_{i,N_i} - t_{i,1})$
mPer	Mean period of all locomotor oscillations	$mPer = \frac{1}{\text{Num}} * \sum_i \sum_j (t_{i,j} - t_{i,j-1})$
PerCV	Coefficient of variation (CV) of locomotor oscillations period	$PerCV = \sqrt{\frac{\sum_i \sum_j \left((t_{i,j} - t_{i,j-1}) - mPer\right)^2}{Num}} / mPer$
Dep	Signal depolarization, magnitude elevation from baseline to stim-on period	$Dep = \text{median}\left(y_{\text{LP}}^{(c)}(t_{stimon})\right) - \text{median}(y_{\text{LP}}^{(c)}(t_{baseline}))$
AUC	Area under the curve of the oscillating signal within locomotor episodes	$AUC = \sum_i \int_{t_{i,1}}^{t_{i,N_i}} y_{\text{LP}}^{(c)}(t)dt$
mWid	Mean width of all locomotor oscillations	$mWid = \frac{1}{Num} * \sum_i \sum_j w_{i,j}$
WidCV	Coefficient of variation (CV) of locomotor oscillations width	$WidCV = \sqrt{\frac{\sum_i \sum_j (w_{i,j} - mWid)^2}{Num}} / mWid$
mPro	Mean prominence of all locomotor oscillations	$mPro = \frac{1}{Num} * \sum_i \sum_j p_{i,j}$
ProCV	Coefficient of variation (CV) of locomotor oscillations prominence	$ProCV = \sqrt{\frac{\sum_i \sum_j (p_{i,j} - mPro)^2}{Num}} / mPro$
Duty	Duty cycle of the oscillating signal within locomotor episodes	$Duty = \frac{mPer}{2 * mWid}$
mLocDev	Mean local deviance of prominence	$mLocDev = \frac{1}{Num} * \sum_i \sum_j l_{i,j}$

N_i: number of oscillations in i-th locomotor episode, $\boldsymbol{y}_{\text{LP}}^{(c)}$: low passed signal.
$t_{i,j}, w_{i,j}, p_{i,j}, l_{i,j}$: timestamp/width/prominence/local deviation of *j-th* oscillation in *i-th* locomotion episode.
$t_{stimon}, t_{baseline}$: timestamps of stim on period and baseline period, as shown in Figure 2B.
